# Supplementary material for: The Systems Biology Research Tool: evolvable open-source software
Source: BMC Syst Biol. 2008 Jun 29;2:55. doi: 10.1186/1752-0509-2-55 (PMC2446383; doi:10.1186/1752-0509-2-55)
Supplement: Additional file 1 — SBRT Archive. An archive of the current version of the Systems Biology Research Tool. [file 1752-0509-2-55-S1.zip › sbrt-1.4.0/doc/developers_guide/api/sbrt/kernel/math/stat/comp/package-use.html]

Uses of Package sbrt.kernel.math.stat.comp


|  |  |  |
| --- | --- | --- |
| **Overview** \| **Package** \| Class \| **Use** \| **Tree** \| **Deprecated** \| **Index** \| **Help** | |  |
| PREV   NEXT | **FRAMES**    **NO FRAMES**     **All Classes** |  |


---


## **Uses of Package sbrt.kernel.math.stat.comp**

| Packages that use sbrt.kernel.math.stat.comp | |
| --- | --- |
| **sbrt.ext.R.stat** | Provides classes that allow communication with R for the purposes of statistics. |
| **sbrt.kernel.math.stat.comp** | Provides classes and interfaces for performing statistical computations. |
| **sbrt.kernel.math.stat.proc** | Provides classes and interfaces for performing statistical processes. |
| **sbrt.shell.mng.stat** | Provides classes and interfaces for creating statistical process managers. |
| **sbrt.shell.text.math** | Provides classes and interfaces for defining formats for mathematical objects. |

Classes in sbrt.kernel.math.stat.comp used by sbrt.ext.R.stat

****Correlator****
  
          This interface is used to represent correlation statistic computers.

****CorrResult****
  
          This interface is used to represent objects that store the results of a
correlation computation.

Classes in sbrt.kernel.math.stat.comp used by sbrt.kernel.math.stat.comp

****Correlator****
  
          This interface is used to represent correlation statistic computers.

****CorrResult****
  
          This interface is used to represent objects that store the results of a
correlation computation.

****KendallTau****
  
          This abstract class is a skeleton implementation of a Kendall's tau
correlation statistic computer.

****TauResult****
  
          This class is used to store the results of a correlation computation.

Classes in sbrt.kernel.math.stat.comp used by sbrt.kernel.math.stat.proc

****Correlator****
  
          This interface is used to represent correlation statistic computers.

****CorrResult****
  
          This interface is used to represent objects that store the results of a
correlation computation.

****MannWhitneyResults****
  
          This class is used to store the results of a Mann-Whitney U test.

Classes in sbrt.kernel.math.stat.comp used by sbrt.shell.mng.stat

****KendallTau****
  
          This abstract class is a skeleton implementation of a Kendall's tau
correlation statistic computer.

****TauResult****
  
          This class is used to store the results of a correlation computation.

Classes in sbrt.kernel.math.stat.comp used by sbrt.shell.text.math

****TauResult****
  
          This class is used to store the results of a correlation computation.

---


|  |  |  |
| --- | --- | --- |
| **Overview** \| **Package** \| Class \| **Use** \| **Tree** \| **Deprecated** \| **Index** \| **Help** | |  |
| PREV   NEXT | **FRAMES**    **NO FRAMES**     **All Classes** |  |


---
